# Alteration of growth factors and neuronal death in diabetic retinopathy: what we have learned so far

**Published:** 2011-01-28

**Authors:** Will Whitmire, Mohammed MH Al-Gayyar, Mohammed Abdelsaid, Bilal K Yousufzai, Azza B El-Remessy

**Affiliations:** 1 Program in Clinical and Experimental Therapeutics, College of Pharmacy, University of Georgia, Augusta, GA; 2 Vision Discovery Institute, Medical College of Georgia, Augusta, GA; 3 Charlie Norwood Veterans Affairs Medical Center, Augusta, GA

## Abstract

**Purpose:**

Diabetic retinopathy (DR) is a leading cause of blindness in American adults. Over the years, DR has been perceived as a vascular disease characterized by vascular permeability, macular edema, and neovascularization that can lead to blindness. Relatively new research on neurodegeneration is expanding our views of the pathogenesis of DR. Evidence has begun to point to the fact that even before vascular complications begin to manifest, neuronal cell death and dysfunction have already begun. Based on the literature and our own studies, we address whether neuronal death is associated with loss of neurotrophic support due to less production of a given growth factor or due to impairment of its signaling events regardless of the level of the growth factor itself.

**Methods:**

In this article we aimed to review the literature that looks at the neuronal side of DR and whether retinal neurons are adversely affected due to the lack of neurotrophic levels or activity. In particular, we examine the research looking at insulin, insulin-like growth factor, vascular endothelial growth factor, pigment epithelium-derived growth factor, brain-derived neurotrophic factor, and nerve growth factor.

**Results:**

Research shows that insulin has neurotrophic properties and that the loss of its pro-survival pathways may have a role in diabetic retinopathy. There is also evidence to suggest that exogenously administered insulin may have a role in the treatment of DR. Insulin-like growth factor has been shown to have a role in retinal neurogenesis and there is early evidence that it may also have neuroprotective effects. While there is evidence of neuroprotective effects of vascular endothelial growth factor, paradoxically, there is also an increased amount of apoptotic activity in retinal neurons despite an increased level of VEGF in the diabetic eye. Further research is necessary to elucidate the exact mechanisms involved. Pigment epithelium derived growth factor has retinal neuroprotective effects and shows evidence that it may be an avenue for future therapeutic use in DR. Brain-derived growth factor has been shown to have neuroprotective effects in the retina and there is also some evidence in diabetic rats that it may have some therapeutic potential in treating DR. Nerve growth factor has also been shown to have neuroprotective effects and research has begun to elucidate some of the pathways and mechanisms through which these effects occur.

**Conclusions:**

Research has shown that there is some degree of neuronal death involved in DR. It is also evident that there are many growth factors involved in this process. Some of these growth factors have shown some potential as future therapeutic targets in DR. These findings should encourage further investigation into the mechanism of these growth factors, their potential for therapy, and the possibility of a new horizon in the clinical care of DR.

## Introduction

Diabetic retinopathy (DR) is the most prevalent diabetic eye disease in the USA. It is the leading cause of blindness in the working age population, affecting 5.3 million adults and causing an estimated 12,000–24,000 new cases of blindness each year [1]. In patients with type I diabetes, 25%–50% show signs of retinopathy after 10–15 years, 75%–95% after 15 years, and 100% after 30 years. Similarly, type II diabetics show higher incidences of diabetic retinopathy with increased duration of disease. Nonproliferative diabetic retinopathy (NPDR) is seen in 23% of type II diabetics after 11–13 years. This number increases to 41% with NPDR after 14–16 years and to 60% after 16 years [2].

### Pathogenesis

While the underlying metabolic pathways of DR are not completely understood, chronic hyperglycemia is thought to be the ultimate cause of the disease [3]. DR results, at least in part, from early damage to the small blood vessels in the retina. To compensate for impaired circulation and ischemia in the retina due to these damaged vessels, neovascularization may occur on the surface of the retina [4]. These newly formed blood vessels as well as the existing damaged capillaries tend to have increased permeability, leading to accumulation of fluid in the macula and decreased visual acuity. Much of the research effort related to DR has been focused on vascular changes, but it is becoming apparent that other degenerative changes beyond the retinal vasculature are occurring that involve the neural retina. These neurodegenerative changes include increased apoptosis of ganglion cells, glial cell reactivity, microglial activation, and altered glutamate metabolism. In 1962, Bloodworth [5] proposed that diabetic retinopathy was not just a disease of the vasculature but was a multifactorial disease involving the neurons and glia of the retina. In agreement, neuronal cell apoptosis [6] and glial dysfunction [7] have been reported in the retinas of diabetic patients.

Neurodegeneration could explain some of the functional visual deficits that begin soon after the onset of diabetes. The electroretinogram is a measure of electrophysiological activity in the retina that measures changes in field potentials elicited by the entire population of retinal neurons. The amplitude of oscillatory potentials as well as deficits in perceptive resolution, such as the ability to discriminate contrast and night vision, were reduced in juvenile individuals with type I diabetes for 5 years or less, before vascular retinopathy had developed [8]. Other studies have demonstrated in diabetic patients and diabetic rats and mice that diabetes induces early and significant increases in apoptotic death of neurons within the inner retina, further supporting the idea of neurodegeneration in DR [5,6,9-16]. Together these data support the notion that diabetes compromises neuronal survival and function in the retina and causes early impairments in vision that precede the detectable vascular lesions associated with DR. Studies examining the process of neurodegeneration have provided multiple potential mechanisms. Metabolic factors that lead to this neuronal cell death have been suggested to include loss of insulin-mediated trophic support [6,17,18] or injury due to accumulation of excess hexosamines [19], tumor necrosis factor-α [20,21], or glutamate (for review see [22]). Mounting evidence also suggests that diabetes-induced oxidative stress contributes to the pathogenesis of neuronal degeneration. Data show that treatments targeting formation of reactive oxygen species and peroxynitrite exert neuroprotective effects in vitro and in vivo [13-16,21,23].

### Release of growth factors

Following acute retinal injury or chronic neuronal stress in diabetes, glial cells, including microglial and macroglial cells (astrocytes and Müller), are activated to protect and repair retinal neurons [24]. Glial activation results in release of growth factors, including some that promote survival and some that promote death of neuronal cells [25,26]. Vascular endothelial growth factor (VEGF) [27], pigment epithelium-derived growth factor (PEDF) [28], transforming growth factor-β (TGFβ) [29], brain-derived neurotrophic factor (BDNF), and nerve growth factor (NGF) [15] are among the trophic factors released by the Müller cells. In the following sections we will look more closely at insulin, insulin-like growth factor (IGF), VEGF, PEDF, BDNF, and NGF and their relationship to both neurodegeneration and DR. More specifically we will look at whether neuronal death is associated with loss of neurotrophic support due to less production of a given growth factor or due to impairment of its signaling events regardless of the level of the growth factor itself.

### Insulin

It is widely accepted that one of the most significant roles of insulin is to stimulate glucose uptake from the blood by peripheral tissues, leading to a reduction of glucose levels in the circulation. The research done in the Diabetes Control and Complications Trial (DCCT) showed that exogenous insulin administration leading to tight glycemic control resulted in decreased incidence and progression of diabetic retinopathy [3,30-32]. More recently, investigators began to examine whether the role of insulin went beyond its effects on blood glucose levels alone. In 1998, Barber et al. [6] found that exogenous insulin given systemically reduced the number of neuronal apoptotic cells in the retina, which suggested a neurotrophic action of insulin. That notion was supported by previous reports showing neurotrophic properties of insulin within the central nervous system independent of blood glucose levels [33,34] and in retinal ganglion cell (RGC) cultures [35]. From there, interest has grown and studies have looked more closely at the action and mechanism of insulin and its receptors in the retina.

While limited research has been performed on vitreous insulin levels, there has been more substantial research into the effects of insulin as a neurotrophic agent. In 2001, Barber et al. demonstrated anti-apoptotic effects of insulin on neonatal rat retinal neurons via activation of the phosphotidylinositol 3 kinase/AKT PI 3-kinase/Akt pathway and inhibition of caspase-3 [17]. Further research demonstrated neurotrophic effects of insulin via other pathways [36,37], and results showed that the retina expresses an equal amount of insulin receptor protein with similar kinase activity as the brain and the liver [37]. In their research Reiter et al. showed that diabetes reduces basal insulin receptor kinase activity and reduces insulin receptor substrate-1/2-associated PI3-kinase/Akt activation in the short-term (4 weeks), with the additional reduction of constitutive insulin receptor autophosphorylation and insulin receptor substrate-2 expression in the long-term (12 weeks) [37]. In the same study it was also shown that both systemic and intravitreal insulin administration restored deficient insulin receptor signaling [37]. This suggests that the loss of the prosurvival insulin-signaling pathway may play a role in diabetic retinopathy. It also points to the possibility that exogenous insulin may have a role in treatment of diabetic retinopathy via its neurotrophic actions. More recent studies show other potential mechanisms by which insulin affects DR, including the regulation of α- and γ-crystallins, factors potentially involved in the inflammatory process in diabetic retinopathy [38]. Due to the potentially hypoglycemic effects of large amounts of systemic insulin required to affect the retina, slow release local delivery of insulin using subconjunctivally implanted hydrogels in rats has proven safe and well tolerated [39]. Further investigation examining both the safety and the efficacy of locally administered insulin in humans would be helpful.

### Insulin-like growth factor

The role of IGF on diabetic retinopathy has been difficult to assess. While its angiogenic effects and its role in neovascularization in DR have been well documented, its role on retinal neurons has not been fully elucidated (reviewed in [40,41]). IGF presents in two forms: IGF-I and IGF-II, with most of the insulin-like effects coming from IGF-I. There are receptors for each IGF homolog, appropriately named IGF-I receptor (IGF-IR) and IGF-II receptor (IGF-IIR), which are found throughout the neuroretinal layers, retinal pigment epithelial, and retinal capillary endothelial cells [42]. Multiple studies have shown that the levels of IGF-I, IGF-II, and insulin-like growth factor binding proteins (IGFBPs) are elevated in the vitreous of patients with PDR [43-45].

The postulated neuroprotective action of IGF is supported by previous studies showing its role in retinal neurogenesis (reviewed [46-48]). There is also evidence of a neuroprotective effect of IGF in retinal ganglion [49,50] and amacrine cells [17]. One study demonstrated the neuroprotective effects of early treatment with systemic IGF-I in diabetic rats [51]. Further studies into the neuroprotective effects of IGF are warranted for helping us better understand whether IGF has a place as a therapeutic target in DR.

### Vascular endothelial growth factor and neuroprotection

The VEGF family incorporates five structurally related ligands (A–D and placenta growth factor [PlGF]) that bind differentially to three receptor tyrosine kinases (VEGF receptor-1, 2, and 3). VEGF-A (also referred to as VEGF) is the founding member and the most characterized member of the VEGF family for its angiogenic and permeability effects. In contrast, VEGF-B is less characterized and its biologic function as a survival factor remains debatable [52,53]. Previous studies showed that VEGF-A binds to VEGFR-1 and 2, while VEGF-B binds mainly to VEGFR-1, which may explain the properties of each regarding vascular permeability, angiogenesis, and survival [54,55]. In the adult retina, VEGF-A has been shown to be produced by retinal pigment epithelium (RPE), endothelial cells, pericytes, astrocytes [56], Müller glial cells, amacrine, and ganglion cells [57]. VEGF-B was found to be expressed in the lens, sclera, retina, iris, and vitreous fluid of the nondiabetic eye [58].

Over the last decade, evidence has been accumulating that VEGF plays a nonvascular and neuroprotective role in adult normal retinas [59]. D’Amore’s group showed that VEGF-A neutralization does not affect normal retinal vasculature but it can cause a neuroretinal cell apoptosis and loss of retinal function [60]. The latter group also showed that the VEGFR-2 receptor is expressed in retinal neuronal tissue (ganglion cell layer [GCL] and inner nuclear layer [INL]), that VEGF is a direct survival factor for photoreceptors, and that VEGF plays a role in Müller cell survival through an autocrine-signaling pathway in nondiabetic models [60]. Studies also have shown that treatment with VEGF-B protects RGC in various models of neurotoxicity [53] as well as retinal vasculature [61]. The neuroprotective effect of VEGF-B was attributed to inhibition of pro-apoptotic proteins, including p53 and members of the caspase family, via the activation of VEGFR-1. These studies suggest that VEGF-B is the first member of the VEGF family that has a potent anti-apoptotic effect, while lacking a general angiogenic activity. Of note, there is no evidence for the neuroprotective effects of VEGF in diabetic human or animal studies.

Paradoxically, while there is an abundance of VEGF-A in the diabetic retina, there is still accelerated vascular and neuronal apoptosis in experimental and clinical samples. A possible explanation might be drawn from our previous studies showing that excessive levels of peroxynitrite produced during diabetes can inhibit the VEGF-mediated survival signal via tyrosine nitration and subsequent inhibition of key survival proteins, the p85 regulatory PI3-kinase in retinal cells [62,63]. The results also indicate that although the oxidative and pro-inflammatory diabetic milieu stimulates VEGF levels, it can also switch its signal from survival to the apoptotic pathways. Further studies are warranted to examine the role of peroxynitrite in inhibiting VEGF’s survival signal in neuronal retinal cells. Another possible explanation for this paradox in diabetic patients is that levels of VEGF-A are increased at the expense of the survival factor VEGF-B, suggesting that VEGF splicing was switched from an anti-angiogenic to a pro-angiogenic environment [58].

Due to the detrimental vascular effects of VEGF in DR, the off-label use of anti-VEGF therapies alone or in combination with laser photocoagulation showed short-term beneficial effects (reviewed in [64]). Current anti-VEGF agents include pegaptanib, ranibizumab, and bevacizumab. Ranibizumab and bevacizumab are humanized monoclonal antibodies that block all VEGF isoforms, while pegaptanib, an aptamer, only blocks the VEGF-A isoform [65]. So far, clinical trials using anti-VEGF treatment focused only on studying the systemic side effects, such as cardiovascular, hypertension, proteinuria, or bleeding [64,66-69] but not the incidence of retinal neurodegeneration, such as retinal atrophy or thinning, or RPE degeneration. Therefore, there is a great need for more studies to fill the scientific gap of the long-term effects of anti-VEGF therapy, especially in diabetic populations.

### Pigment epithelial derived factor

PEDF is a neurotrophic factor that occurs naturally in the eye and is expressed in multiple retinal cells, including retinal pigment epithelial cells [70], glial cells, vascular endothelial cells, Müller cells, and neurons [71]. PEDF was originally identified based on its ability to induce differentiation of retinoblastoma cells but has subsequently been recognized as a neurotrophic and angiostatic growth factor [70,72,73]. Studies showing decreased levels of PEDF in ocular fluids and vitrectomy specimens from patients with diabetic retinopathy suggest that the loss of PEDF contributes to diabetes-induced neuroglial cell toxicity [70,71]. In the same patients there was an inverse correlation between elevated VEGF expression and decreased levels of PEDF, suggesting that a shift in the balance between levels of PEDF and VEGF may contribute to the development of retinal neovascular disease [70]. Recent studies with cultured cells indicate that hypoxia and VEGF downregulate levels of PEDF by increasing the activity of matrix metalloproteinase enzymes, which degrade and inactivate PEDF [74]. Moreover, it has been shown that PEDF can also reduce oxidative stress by suppressing reduced form of nicotinamide adenine dinucleotide phosphate (NADPH) oxidase-mediated generation of reactive oxygen species [75,76].

PEDF can influence both cell differentiation and survival of neurons in the brain, eye, and spinal cord (reviewed in [70]). PEDF has retinal neuroprotective effects where it can prevent ischemic damage to photoreceptors and dopaminergic neurons [70,77]. Furthermore, pretreatment of retinal photoreceptor cultures with PEDF significantly increased cell survival in vivo [78,79] and in vitro [80] models of oxidative stress and light damage. These promising results suggest that enhancing the expression and function of this protein can be a therapeutic target in DR. The neuroprotective role of PEDF, however, has not been examined in models of diabetes, a disappointment that should encourage further studies on the role of PEDF.

### Neurotrophins

The neurotrophins (NTs) are structural families of secreted proteins that have potent effects on neuronal differentiation, survival, neurite outgrowth, synaptic formation, and plasticity [81,82]. Neurotrophins are initially synthesized in a proform that is cleaved by Ca^2+^-dependent serine endoprotease belonging to the subtilisin-like proprotein convertase (SPC) family including furin [83] and plasmine [84], to release the mature NT form. The neurotrophins are the preferred ligands for tropomysine like kinase (Trks) receptors, while they can only activate P75 neurotrophin receptor (p75^NTR^) in low-affinity-binding configurations [85,86]. Signals emanating from Trks support neuronal survival, growth, and synaptic strengthening, while those emanating from p75^NTR^ induce neuronal apoptosis, attenuate neurite outgrowth, and weaken synaptic signaling [85,86].

### Nerve growth factor

NGF is the first discovered and best characterized member of the growth factor family [87]. NGF is not only an important regulator of retinal development but also plays a key role in regeneration of neural circuits in the visual system in retinal degenerative diseases [88]. The relevance of NGF to diabetic retinopathy was first demonstrated by the study of Hammes et al. [10]. They showed that the treatment of diabetic rats with NGF prevented both early apoptosis of neuronal death in RGC and Müller cells as well as the development of pericyte loss and acellular occluded capillaries [10]. These results suggested that diabetes might reduce the level of the main neurotrophic factor NGF, thus causing the complication. However, several reports documented paradoxical increases in NGF levels in the serum of patients with insulin-dependent diabetes mellitus [89,90] and in the serum and tears of patients with diabetic neuropathy and retinopathy [89,91-94]. The increases of NGF levels positively correlated with the diabetic retinopathy stage and other diabetes mellitus (DM) parameters [91].

NGF is synthesized and secreted by glia or microglia [95] as a precursor (proNGF). It is then proteolytically cleaved intracellularly by furin and extracellulary by matrix metalloproteinase-7, generating the mature form (NGF) [96]. Until recently our knowledge about the release of neurotrophins in diabetic tissue had been limited to techniques, such as enzyme-linked immunosorbent assays (ELISA) and quantitative measurement of mRNA expression, that could not differentiate proNGF from mature NGF. While mature NGF mediates neuronal cell survival through binding TrkA and p75^NTR^ receptors [88], proNGF can promote neuronal apoptosis because of its high affinity to p75^NTR^ [97]. Under oxidative stress and inflammatory conditions, the activity of proteases is altered, which can result in accumulation of proNGF in injured neuronal and vascular tissues [98]. However, the homeostasis of NGF and proNGF levels within the diabetic eye remained elusive. Our recent studies demonstrated significant and progressive increases in levels of proNGF at the expense of NGF in human samples from diabetic patients, PDR patients, and experimental diabetes [15]. Our studies demonstrated also that Müller cells are the major source of proNGF synthesis in response to high glucose or peroxynitrite [16]. Thus, peroxynitrite activates Müller cells to secrete proNGF and then impairs its maturation by inhibiting matrix metalloproteinase-7, leading to accumulation of proNGF and reduction of mature NGF. As expected, the lack of neurotrophic support was associated with retinal neurodegeneration and in particular RGC cells. To add another level of complexity, the diabetic and pro-oxidative milieu not only can affect the homeostasis of the NGF but can also alter the expression and function of its receptors—the survival TrkA receptor and the neurotrophin p75^NTR^ receptor. Although TrkA expression is not altered, the phosphorylation of the receptor and hence its activity is impaired via tyrosine nitration in experimental [16] and clinical retina samples [13,15]. On the other hand, diabetes causes significant upregulation of retinal p75^NTR^ expression in clinical and experimental diabetes [13,15,16]. Our recent studies further elaborated on the critical role of p75^NTR^ in mediating RGC death via activation of the pro-apoptotic p38 mitogen-activated protein kinase (p38MAPK) pathway, resulting in retinal neurodegeneration in clinical and experimental diabetes [13,15,16]. The later study indicated that upregulation of p75^NTR^ can play a key role in glial activation and release of proNGF under diabetic conditions. Further studies are in progress to better understand the role of p75^NTR^ in retinal neuroglial inflammation and to characterize the underlying signaling pathway in hopes of identifying potential therapeutic targets for diabetic retinopathy.

### Brain-derived neurotrophic factor

BDNF is expressed in several retinal cells, including RGC and Müller glia in the retina, and has been previously reported to prevent RGC and amacrine cell death [99]. Although many studies have described the important roles of BDNF in the physiology and pathophysiology of the retina, few studies have examined the changes of BDNF levels or activity in models of diabetic retinopathy. One study demonstrated significant reduction (approximately 50%) of the mRNA and protein expression level of BDNF in diabetic rat retina that were positively correlated with degeneration of dopaminergic amacrine cells accompanied by a reduction in BDNF levels [100]. Similar reduction of BDNF levels were observed using diabetic mice that were associated with impaired visual function [101]. Furthermore, the first study also demonstrated the therapeutic potential of BDNF using multiple intraocular injections for treating neurodegeneration in the diabetic rat retina [100].

In summary, we reviewed the literature that looks at the neuroglial activation side of DR and the subsequent release of growth factors. More specifically we looked at whether diabetes adversely affects retinal neurons due to lack of neurotrophic levels, such as in the case of insulin, PEDF, and BDNF, and/or due to lack of activity, such as in the case of VEGF and NGF. To date, a limited number of studies have assessed the expression of growth factors and how they can affect various retinal neurons in response to experimental diabetes (Table 1). Among research dealing with retinal neurons, RGCs are the most studied, and several studies did not specify the type of retinal neurons and instead looked simply at neuronal versus vascular effects. Such findings call for further investigation into retinal neurodegeneration and the alteration of growth factors, their potential for therapy, and the possibility of a new horizon in the clinical care of DR.

**Table 1:** Summary of reports that examined growth factors in relation to various neuronal cells under diabetic condition.

**GF**	**Model**	**Summary**	**Cell type**	**References**
Insulin	STZ	Insulin provides trophic support for retinal neurons via PI 3-kinase/Akt-dependent pathway.	Neuronal/RGC	[6,17,37]
IGF-1	STZ	Systemic IGF-I reduced neuroretinal cell death.	Photoreceptors	[51]
VEGF	STZ	Elevated VEGF causes vascular dysfunction, which was associated with neuronal death.	Neuronal	[21,102]
PEDF	STZ	PEDF prevented neuronal derangements and restored electroretinal gram function.	Neuronal	[103]
BDNF	STZ	BDNF provides trophic support for retinal neurons and amacrine cells.	Neuronal/amacrine	[100,101]
NGF	Human/STZ/culture	Restoring NGF level and signal prevented retinal neuronal/RGC death.	Neuronal/RGC	[10,13,15,16,91]

Abbreviations: STZ represents streptozocin, IGF represents insulin-like growth factor, VEGF represents vascular endothelial growth factor, PEDF represents pigment epithelium-derived growth factor, BDNF represents brain-derived neurotrophic factor, NGF represents nerve growth factor, RGC represents retinal ganglion cell.
